# Supplementary material for: ProteinShader: illustrative rendering of macromolecules
Source: BMC Struct Biol. 2009 Mar 30;9:19. doi: 10.1186/1472-6807-9-19 (PMC2672931; doi:10.1186/1472-6807-9-19)
Supplement: Additional file 1 — ProteinShader program without source code. This compressed file contains the complete ProteinShader program including associated libraries, but no source code. A README.txt file gives an overview of the ProteinShader distribution, and the index.html file in the help subdirectory has directions on getting started with the program as well as a set of tutorials. [file 1472-6807-9-19-S1.zip › ProteinShader-beta-0_9_4-binary/help/api/org/proteinshader/gui/utils/package-tree.html]

org.proteinshader.gui.utils Class Hierarchy (ProteinShader API)


|  |  |  |
| --- | --- | --- |
| **Overview** \| **Package** \| Class \| Use \| **Tree** \| **Deprecated** \| **Index** \| **Help** | |  |
| **PREV**   **NEXT** | **FRAMES**    **NO FRAMES**     **All Classes** |  |


---


## Hierarchy For Package org.proteinshader.gui.utils

**Package Hierarchies:**: All Packages

---

## Class Hierarchy

- java.lang.**Object**
  - java.awt.**Component** (implements java.awt.image.ImageObserver, java.awt.MenuContainer, java.io.Serializable)
    - java.awt.**Container**
      - javax.swing.**JComponent** (implements java.io.Serializable)
        - javax.swing.**JFileChooser** (implements javax.accessibility.Accessible)
          - org.proteinshader.gui.utils.**ImageFileChooser**
          - javax.swing.**JLabel** (implements javax.accessibility.Accessible, javax.swing.SwingConstants)
            - javax.swing.**DefaultListCellRenderer** (implements javax.swing.ListCellRenderer, java.io.Serializable)
              - org.proteinshader.gui.utils.**CenteredListCellRenderer**- org.proteinshader.gui.utils.**PaddedListCellRenderer**- org.proteinshader.gui.utils.**SeparatorListCellRenderer**
    - javax.swing.filechooser.**FileFilter**
      - org.proteinshader.gui.utils.**FileExtensionFilter**
      - org.proteinshader.gui.utils.**FpsLabel**

---


|  |  |  |
| --- | --- | --- |
| **Overview** \| **Package** \| Class \| Use \| **Tree** \| **Deprecated** \| **Index** \| **Help** | |  |
| **PREV**   **NEXT** | **FRAMES**    **NO FRAMES**     **All Classes** |  |


---

# *Copyright © 2007-2008*
